# Anticentromere antibody induced by immunization with centromere protein and Freund’s complete adjuvant may interfere with mouse early-stage embryo

**DOI:** 10.1186/s12958-021-00813-1

**Published:** 2021-08-20

**Authors:** Hanyan Liu, Yufen Zhang, Haiying Liu, Qing Huang, Ying Ying

**Affiliations:** 1 Department of Obstetrics and Gynecology, Center for Reproductive Medicine, Key Laboratory for Major Obstetric Diseases of Guangdong Province, The Third Affiliated Hospital of Guangzhou Medical University, Guangzhou, China; 2 Department of Obstetrics and Gynecology, Fengshun County People’s Hospital, Fengshun county, Meizhou City, Guangdong Province China

**Keywords:** Anticentromere antibody, Early-stage embryo

## Abstract

**Background:**

Anticentromere antibody (ACA) is a member of the antinuclear antibody spectrum (ANAs) which has been speculated to be associated with subfertility. Thus, the present study aimed to investigate the induction of ACA production and its potential interference with early-stage embryos.

**Methods:**

Recombinant centromere protein-A (CENP-A) or centromere protein-B (CENP-B) and complete Freund’s adjuvant (CFA) were used to immunize mice. Serum ACA level was then evaluated by using an indirect immunofluorescence test. Immunofluorescence assay was performed to detect IgG in follicles in ovarian tissues and early-stage embryos.

**Results:**

Following treatment, serum positive ACA was observed in mice treated with CENP and CFA. Furthermore, IgG were detected in follicular fluid and early-stage embryos from mice treated with CENP and CFA.

**Conclusions:**

This study preliminarily indicated that ACA induced by CENP and CFA may penetrate into the living embryos of early-stage in mice.

## Background

Anticentromere antibody (ACA), a member of the antinuclear antibody spectrum (ANAs), is regarded as an important autoimmune serological marker for systemic sclerosis (SSc), particularly the form of SSc known as CREST (calcinosis cutis, Raynaud’s phenomenon, esophageal dysfunction, sclerodactyly and telangiectasia) syndrome [1, 2]. The association between ACA and infertility has been discussed in recent papers [3–5].

Centromere protein-A (CENP-A) and -B (CENP-B) are constitutive proteins in the complex centromere protein system, and their crucial role in centromere assembly and function has been studied intensively [6]. CENP-A and CENP-B are the major antigens for ACA in patients with SSc [7, 8]. Human ACA is able to identify and bind to the centromere/kinetochore complex in vertebrates, invertebrates and plants, indicating that the antigenicity of the centromere among different species is highly conserved across evolution [9–12].

Thus, in the present study, recombinant human CENP and complete Freund’s adjuvant (CFA) were used to induce ACA production, and the impact of induced ACA on early-stage embryos was evaluated. Therefore, the purpose of this study was to preliminarily investigate whether ACA could enter the living early-stage embryos in mice.

## Methods

### Mice

Six-week-old wild-typefemale C57BL/6J mice were purchased from Guangdong Medical Laboratory AnimalCenter (Guangzhou, China). All the experiments and procedures were approved bythe Ethics Committee of the Third Affiliated Hospital of Guangzhou MedicalUniversity (Guangzhou, China).

### CENP and CFA treatment

Recombinant human CENP-A orCENP-B (catalog nos. orb81023 and orb81024, respectively; Biorby Ltd.,Cambridge, UK) were solubilized in saline (forming HA and HB solution,respectively). HA or HB solution was mixed 1:1 (volume/volume) with completeFreund’s adjuvant (CFA, Sigma-Aldrich, Merck KGaA, Darmstadt, Germany). Thesesolutions (200 μl, containing 100 μg HA or HB) were injected subcutaneously atthe same site on the shaved back of the mice with a 26-gauge needle three timesat an interval of 2 weeks. Mice subcutaneously injected with CFA or saline wereused as controls. The mice were categorized into four groups (*n*=6/group)according to treatments: HA/CFA group, HB/CFA group, CFA group and salinegroup.

### Mouse early-stage embryoscollection

Superovulation was performed2 weeks after the last drug injection using pregnant mare serum gonadotrophin(PMSG, 10 IU, i.p) and human chorionic gonadotrophin (HCG, 10 IU i.p after 48h), mice from each group were mated 1:1 with male mice. Subsequently, after 24h, the female mice with the plugs were separated and sacrificed by cervicaldislocation, and the fertilized oocytes were collected by sharp dissection ofthe fallopian tube and transferred to the cleavage stage culture medium for invitro culture.

### Determination of mouseanti-CENP-A and mouse anti-CENP-B antibodies in the serum

Mouse serum anti-CENP-A andanti-CENP-B antibodies were assessed using an indirect immunofluorescence test(IIFT) kit for antinuclear IgG antibodies (IIFT Mosaic: HEp-2/Liver [Monkey];cat. no. FA 1510-1003-1, Euroimmun AG, Luebeck, Germany). IIFT is the standardassay for the determination of antibodies against nuclear antigens. Since thesample to be tested was mouse serum, we substituted the anti-human secondaryantibody in the original kit with the Alexa Fluor 488-conjugated goatanti-mouse IgG (Cell Signaling Technology, USA).

### Immunofluorescence assayfor the determination of IgG in follicle of ovarian tissue and in early-stageembryos

Ovarian tissue sections wereincubated for 1 h with red fluorescein labeled anti-mouse IgG(115-165-003,Google Biology, Wuhan, China). Following washing to remove theexcess conjugate, the sections were stained with DAPI and visualized under afluorescent microscope (Olympus BX61; Olympus Corporation, Tokyo, Japan).

Fertilized oocytes were cultured in Quinn’s series medium (SAGE, USA). Three embryos from each group were selected for 3 consecutive days and incubated for 2 h with fluorophore-labeled donkey anti-mouse IgG (H + L) antibody (1:1,000 dilution; cat. no. A21202; Invitrogen; Thermo Fisher Scientific, Inc.). Following washing to remove the excess conjugate, the presence of fluorescence was examined using a laser scanning confocal microscope (LSM780; Zeiss GmbH, Jena, Germany).

### Statistical analysis

 Statistical analysis was performed using SPSS 13 (SPSS, Inc., Chicago, IL, USA). All continuous variables were expressed as the mean ± standard deviation. The Mann-Whitney U test was used to evaluate differences among groups, and analysis of variance followed by Bonferroni adjustment was used for multiple comparisons. *P* < 0.05 was considered statistically significant.

## Results

### Serum positive ACA in mice treated with CENP and CFA

**Fig. 1:**
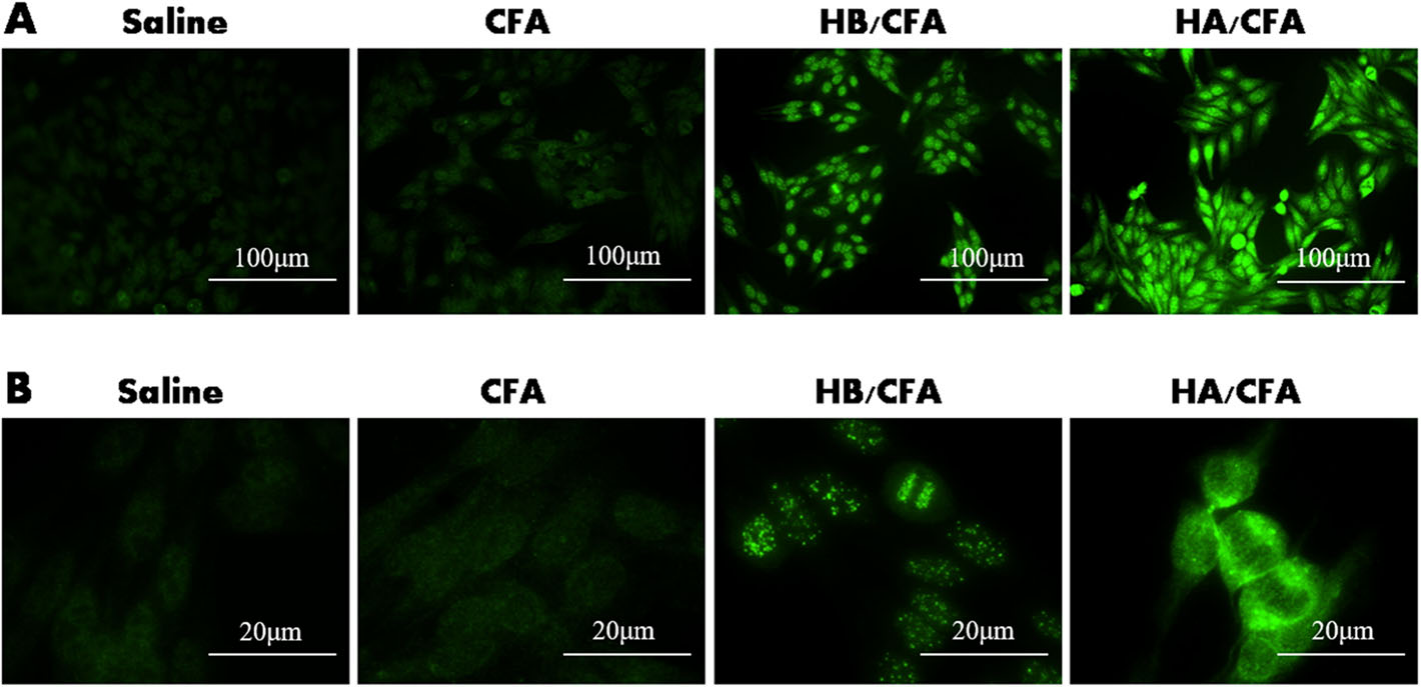
Indirect immunofluorescence test to determine ACA in mouse serum after 6 weeks of treatment. Positive ACA was detected in serum samples from the HA/CFA and HB/CFA groups, which exhibited a typical centromeric-type fluorescence pattern of discrete punctate staining in the nucleus, while none of the serum samples from the CFA and saline groups showed fluorescence signal (*n* = 6 for each group). **A** and **B** are representative images with an original magnification ×200 and ×1000, respectively

Treatment with CENP-A andCFA or CENP-B and CFA induced the production of ACA in mice. Theimmunofluorescence assay exhibited a positive nuclear staining in the serumsamples from the HA/CFA and HB/CFA groups; none of the serum samples from theCFA and saline groups exhibited any fluorescence signal (Fig. 1).

### Accumulation of IgG in follicular fluid induced by CENP andCFA treatment

**Fig. 2:**
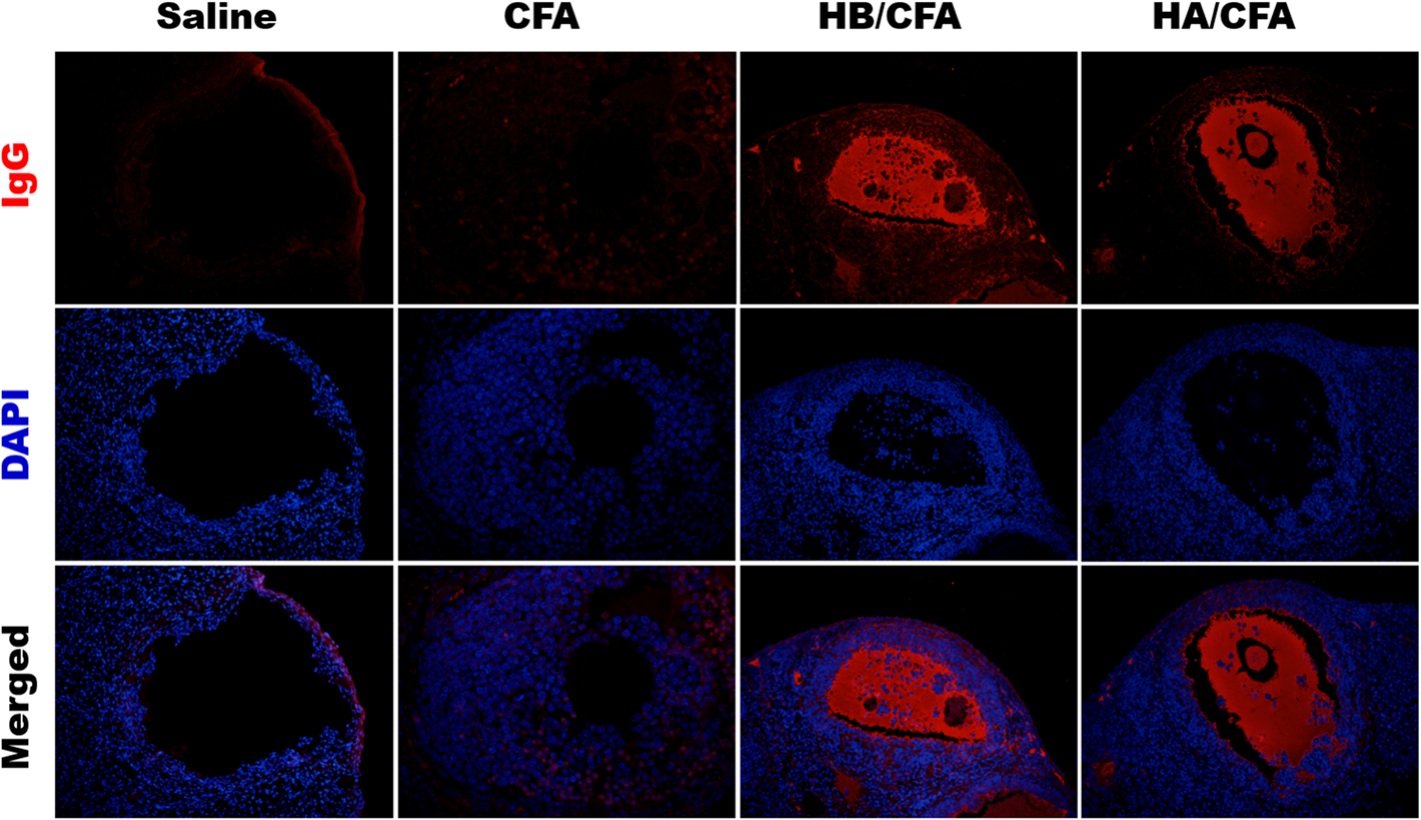
Accumulation of IgG in follicular fluid induced by CENP and CFA treatment (original magnification ×200). The red fluorescence of IgG, predominantly distributed in the follicular fluid of ovarian tissue section, was observed in mice from the HA/CFA and HB/CFA groups. No fluorescence of IgG was visualized in mice from the CFA and saline groups

 Immunofluorescenceassay of ovarian tissue section showed strong fluorescence of IgG in follicular fluid in micefrom both the HA/CFA and HB/CFA groups. However, no fluorescent signal wasvisualized in the follicular fluid from mice treated with CFA or saline (Fig. 2).

### IgG fluorescence in early-stage embryos are observed followingtreatment with CENP and CFA

**Fig. 3:**
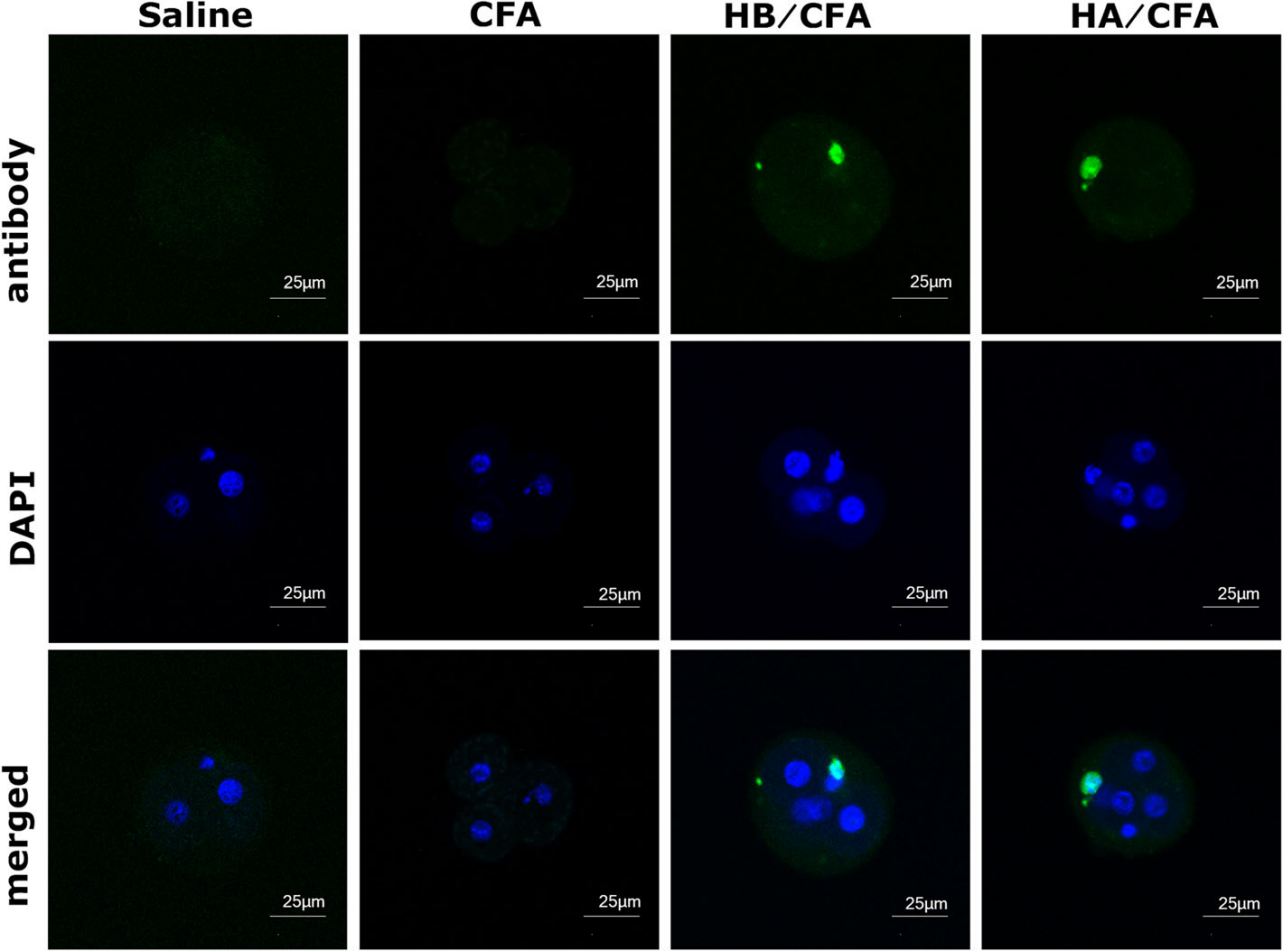
Immunofluorescence assay to determine the antibody in early-stage embryos (original magnification ×400). Green fluorescence of antibody distributed only on nucleus was visualized in early-stage embryos from HA/CFA and HB/CFA groups, which could be inferred from the overlap of green fluorescence of antibody and blue fluorescence of DAPI staining. None of the embryos from the CFA or saline groups showed the fluorescence of antibody

All embryos from micetreated with CENP-A and CFA or CENP-B and CFA exhibited fluorescence of IgGdispersed in the nucleus. This phenomenon was based on the overlap of greenfluorescence of antibodies and the blue fluorescence of DAPI staining. However,none of the embryos from mice treated with CFA or saline exhibited anyfluorescence of antibodies (Fig. 3).

## Discussion

In the current study, mice treated with CENP and CFA not only exhibited higher levels of ACA in the serum, but also exhibited a large quantity of IgG in the follicular fluid. We speculate that the majority of immunoglobulins in the follicular fluid could be ACA, and the potential impact of these IgG on oocytes and early-stage embryos should be considered in further studies.

It was reported that different types of ANAs could enter living cells [13, 14]. Nevertheless, there is little evidence to suggest that ANAs could enter the oocyte or the embryo. In 1999, researchers identified that early-stage mouse embryos cultured with purified IgG from ANA-positive serum exhibited strong fluorescence of antibodies and experienced significant growth impairment, whereas other types of autoantibodies, such as anti-thyroid and control immunoglobulins, were not able to bind to embryos, suggesting a specific binding between ANAs and embryo [15]. We have identified in our recent study the development and maturation of oocytes were impaired in peripheral ACA positive mice, which exhibited severe chromosomal misalignments in metaphase meiosis, however, no evidence of ACA entering the oocytes was observed, thus the underlying mechanism needs further exploration [16].

However in this study, the results of immunofluorescence assay showed strong immunofluorescence of antibody against nuclear components (which were speculated to be ACA), indicating that mouse embryos may be a direct target for some ACA in vivo prior to implantation.

In addition, for the majority of tested embryos, always only one or some of the blastomeres showed fluorescence. Perhaps, the density of structures in and around the centromere prevents ACA accessibility, or the blastomere with detectable fluorescence was inclined to apoptosis and displayed relatively loose structures that enabled ACA accessibility. However, the precise mechanism needs further clarification.

In the present study, the mechanism by which antibodies entered the living cells has not been elucidated, and the concept of antibodies entering living cells has not been fully defined. However, previous studies have provided some evidence of this. For instance, it was reported that the entry of antibodies into cells was via glycosaminoglycans [17], Fc receptors [18], DNA-histone complexes [19], and myosin 1 [20].

## Conclusions

The presence of ACA induced by immunization with CENP and CFA may penetrate into the living early-stage mouse embryo. The mechanism by which antibodies enter the living cells and the underlying interference mechanism on the early-stage embryos require further exploration.

## Data Availability

The datasets used and/or analyzed in the current study are available from the corresponding author upon reasonable request.

## Abbreviations

ACA: Anticentromere antibody
ANAs: Antinuclear antibodyspectrum
CENP: Centromere protein
CFA: Complete Freund’s adjuvant
CREST: Calcinosis cutis, Raynaud’sphenomenon, esophageal dysfunction, sclerodactyly and telangiectasia
IIFT: Indirect immunofluorescencetest
